# Spatial Mapping of OH Radicals Produced by Electric Discharge in Hydrodynamic Cavitation Cloud

**DOI:** 10.1021/acs.jpclett.5c00979

**Published:** 2025-06-13

**Authors:** Radek Horňák, Jan Čech, Pavel St’ahel, Lubomír Prokeš, David Trunec, Pavel Rudolf, Blahoslav Maršálek

**Affiliations:** † Department of Plasma Physics and Technology, Faculty of Science, Masaryk University, Kotlářská 2, 611 37 Brno, Czech Republic; ‡ 376202 Brno University of Technology, Faculty of Mechanical Engineering, V. Kaplan Department of Fluid Engineering, Technická 2896/2, 616 69 Brno, Czech Republic; ¶ 86891 Institute of Botany of the Czech Academy of Sciences, Lidická 25/27, 602 00 Brno, Czech Republic

## Abstract

As strong oxidizers, OH radicals are crucial for water treatment applications involving plasma–liquid interactions. Hydrodynamic cavitation-based systems show promise for disinfection and micropollutant removal at flow rates of several m^3^/h. Knowledge of the spatial distribution of OH is limited. However, this is vital for enhancing system efficiency. This study maps the spatial distribution of OH generated by electric discharge in a hydrodynamic cavitation cloud. Using Luminol as a chemiluminescent probe, the study addresses challenges related to probe stability and luminescence lifetime in a dynamic cavitation environment. Luminescence decay time was assessed with a fast-frame camera, and spatial mapping was conducted by using an ICCD camera with an optical filter. Strong emission was observed at the collapsing end of the cavitation cloud and within the discharge channel, indicating the production and transport of OH into the liquid.

## Graphical abstract

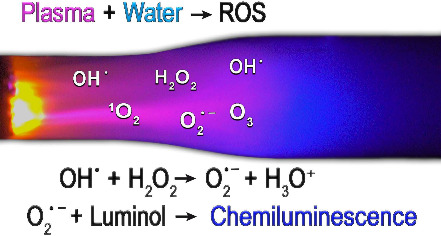

Mapping reactive oxygen species (ROS) is crucial for gaining a thorough understanding and enabling optimization of plasma-based water treatment methods. ROS are the key agents in eliminating micropollutants, but the fluid-plasma interaction and ROS generation are currently treated as a “black box”. The hydroxyl radical (OH^•^) plays an important role among ROS because it is a strong oxidizer and can thus react with most organic substances. Luminol (5-amino-2,3-dihydrophthalazine-1,4-dione, C_8_H_7_N_3_O_2_), a versatile chemiluminescent probe, has been widely employed to detect ROS such as OH^•^, O_2_^•–^, and indirectly also hydrogen peroxide (H_2_O_2_) in diverse aqueous systems, owing to its cost-effectiveness, high sensitivity, and adaptability to both clinical and environmental applications.^1^ The chemiluminescence of Luminol occurs through its alkaline oxidation, producing an excited 3-aminophthalate dianion (C_8_H_5_NO_4_^2–^) that emits blue light at 425 nm upon relaxation. Alkaline conditions, such as those provided by NaOH, are crucial for deprotonating Luminol into its reactive dianionic form (L^2–^), which is more easily oxidized by ROS like OH^•^ and H_2_O_2_ in a more-step process to generate the light-emitting species. This mechanism has been extensively studied using spectroscopic and computational approaches.^2^ Theoretical models by Uchida et al.^3^ analyzed the kinetic interaction between Luminol and H_2_O_2_, highlighting pH-dependent pathways that influence the chemiluminescence intensity. Meanwhile, Lu et al.^4^ examined the challenges of ROS selectivity in Luminol-based assays, focusing on competing reactions involving superoxide (O_2_^•–^) and singlet oxygen (^1^O_2_).

In experimental fluid dynamics, Luminol chemiluminescence has proven indispensable for spatially mapping transient ROS fields, as demonstrated by Lucas et al.,^5^ who visualized ultrasonic cavitation bubbles via OH^•^-driven luminescence. Hydrodynamic cavitation, which uses pressure-induced bubble collapse to generate ROS, has also been studied using Luminol chemiluminescence,^6^ which quantified OH^•^ yields across different cavitation conditions and highlighted the impact of flow turbulence on ROS distribution.

Recent studies on plasma–liquid systems highlight persistent ambiguities in distinguishing OH^•^ from H_2_O_2_ contributions in plasma-treated liquids^7^ and delineate the challenges of tracking ROS transport from gas-phase plasma to bulk liquid, particularly for unstable species like OH^•^.^8^ Shirai et al.^9^ correlated atmospheric-pressure plasma–liquid interactions with chemiluminescence hotspots to resolve short-lived species like OH^•^ and O_2_^•–^. Subsequent work^10^ linked gas-phase OH^•^ density to liquid-phase chemiluminescence intensity, establishing Luminol as a bridge between plasma diagnostics and aqueous ROS chemistry.

The hydrodynamic cavitation-based plasma treatment provides high throughput, while maintaining hydrogen peroxide energy yield and production rates on par with the best-in-class plasma–liquid systems.^11,12^

Despite hydrogen peroxide often being used as a simple comparative marker for liquid–plasma system performance, it is not the most potent reactive oxygen species generated in plasma–liquid systems. This position belongs to the OH^•^ radical, a precursor species of hydrogen peroxide. The knowledge of OH^•^ production rates and its spatiotemporal dynamics is therefore of great interest for the development of advanced oxidation water purification system^13^ or fundamental research of plasma–liquid interaction phenomena.^14^

Various hydrodynamic cavitation-based plasma systems were also reported recently for their promising potential of large-volume applications for micropollutant removal, e.g., pharmaceuticals,^15,16^ or biocontamination remediation, e.g., from cyanobacteria using either direct treatment of contaminated water^17^ or indirect treatment^18^ using plasma-treated water. Knowledge of the ROS dynamics will give necessary insights for further development of these technologies.

The proof of ROS (H_2_O_2_, OH^•^, O_3_) presence in liquid treated using a hydrodynamic cavitation plasma source based on a Venturi nozzle flow geometry with coaxial electrode configuration,^19^ called CaviPlasma, was already reported.^12,20^ In these experiments, OH^•^ was detected using chemical-derivative probes. However, neither the fluorescence spectrometry of terephthalic acid oxidation product^12^ nor the electron paramagnetic resonance spin-trapping technique^20^ were able to disclose the dynamics of the ROS production concerning the particular position within the plasma-cavitation geometry.

In this study, we utilized OH^•^-induced chemiluminescence to map the OH^•^ presence in a hydrodynamic cavitation plasma source. We took advantage of the chemiluminescence of Luminol dissolved in an alkaline solution to follow the spatial dynamics of the OH^•^ radical-induced reactions. Building on prior studies,^5,6,9,10^ we use the catalyst-free Luminol–NaOH system, relying on direct oxidation by plasma-generated OH. While OH^•^ is not the sole radical produced in the gas phase (e.g., O_3_, H_2_O_2_, and O_2_^•–^ are also generated via plasma-chemical reactions^14,21^ ), interfacial processes govern the dissolution of reactive species into the liquid phase.^20,21^ Our focus is on dissolved ROS, as these drive the observed chemiluminescence and are the most relevant to applications. In our recent study^20^ the EPR spectrometry utilizing spin-trapping confirmed the presence of dissolved OH^•^ and ^1^O_2_ via DMPO (5,5-dimethyl-1-pyrroline N-oxide) adducts, while chemical probes quantified long-lived species such as H_2_O_2_ (dominant at ∼1000× higher molar concentration than OH^•^) and O_3_ (∼3% of H_2_O_2_ levels).

The selectivity of Luminol for OH^•^ in our system is supported by the literature and observed kinetics. Both OH^•^ and O_2_^•–^ are involved in Luminol luminescence^3,10,22^ via the two-step mechanism. The Luminol negative ion is oxidized by OH^•^, and then the excited state is obtained by reaction with O_2_^•–^. Shirai et al.^10^ suggest that O_2_^•–^ in liquid phase is the product of OH^•^ reacting with H_2_O_2_ (OH^•^ + H_2_O_2_ → O_2_^•–^ + H_3_O^+^). Given the measured 4:1000 ratio of OH^•^:H_2_O_2_ in CaviPlasma treated liquid,^20^ O_2_^•–^ production is limited by dissolved OH^•^. The observed luminescence decay follows the pseudo-first-order kinetics expected for high concentration of Luminol (1 mM) reacting with low-concentration of OH^•^ (∼10 μM).^12^ This supports OH^•^ as the primary driver of the observed signal. Furthermore, the rapid decay of luminescence (∼0.1 ms after exitation) was reported.^10,22^

We also observed no increase in luminescence signal with treatment time; i.e., the accumulation of long-lived reactive species does not increase the luminescence signal. This further supports our assumption that only short-lived species generated in the discharge act as triggers of the studied chemiluminescence.

Our approach bridges insights from atmospheric-pressure plasma diagnostics^7,10^ and hydrodynamic cavitation chemistry,^6^ offering real-time visualization of ROS dynamics in a continuous flow configuration.

The study required the presence of a chemical probe in the active plasma zone. The terephthalic acid adopted to probe the OH^•^ presence in the plasma-treated liquid was suspected to be prone to decomposition in the discharge (see our recent study).^12^ Therefore, plasma-treatment tests with Luminol solution were performed. The Luminol was used in excess to eliminate the effect of direct degradation of Luminol itself. Our analysis focuses on relative luminescence intensity (spatial/temporal trends) rather than absolute quantification, which minimizes the impact of Luminol degradation on the interpretation of ROS dynamics. Despite the expected degradation of Luminol in the active plasma zone, chemiluminescence was still clearly observable. This manifested as a long, blue-glowing tail extending several centimeters downstream of the collapse zone of the hydrodynamic cavitation cloud (HCC) (see Figure 1 top image). The scheme of the experimental setup is also shown in Figure 1 bottom image, which also illustrates the discharge configuration and associated luminescence.

**Figure 1:**
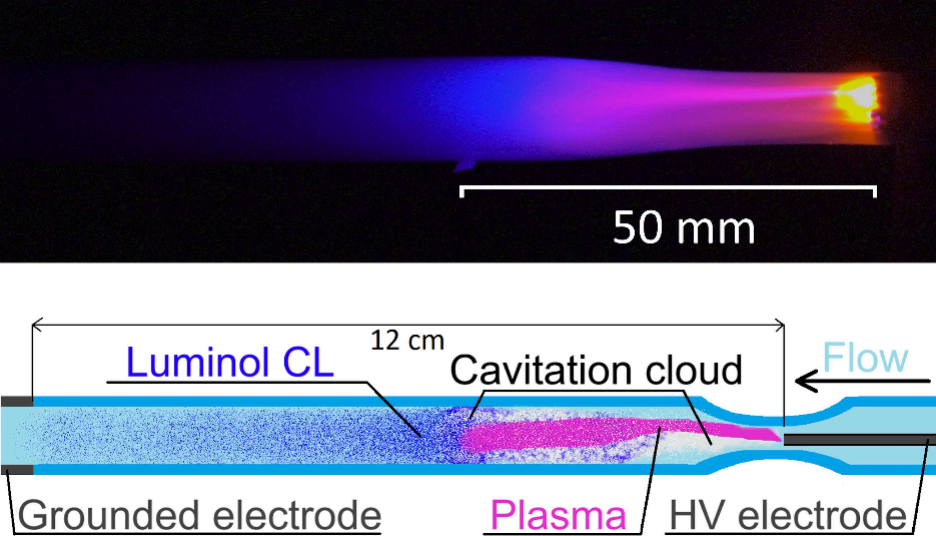
CaviPlasma discharge generated in alkaline Luminol/NaOH solution. Top: the visual appearance at image exposure time of 33.3 ms. Flow rate was set to 0.94 m^3^/h. Flow direction from the right to the left. Bottom: schematic representation of hydrodynamic cavitation discharge tube.

The reported chemiluminescence spectrum of Luminol consists of a broad emission band peaking around 425 nm,^10^ enabling detection using standard non-UV acquisition techniques. The emission spectrometry performed along the discharge axis confirmed the reported spectral pattern of the chemiluminescence signal to be present in the discharge and behind HCC, except the edge of the nozzle electrode. Unfortunately, in the active discharge zone, the discharge emission, i.e., the Balmer hydrogen lines (particularly the H_γ_ at 434 nm) and a weak tail of a UV continuum of molecular hydrogen, overlapped the luminescence signal, preventing the straightforward 2D interpretation of the visually observed emission signatures. To isolate the luminescence signal in the active discharge zone, two approaches were adopted: (i) the optical imaging of discharge using the interference filter and (ii) the spatiotemporal observation of the luminescence decay after discharge extinction.

The results of the filtered optical imaging luminescence emission are given in Figure 2. In order to achieve a high dynamic range and low noise floor, an intensified CCD camera (ICCD) with 16-bit digitization was used, equipped with a telelens with a front-mounted optical interference filter. The 10 nm full width at half maximum filter centered at 420 nm was selected from the available range of filters, enabling the cutoff of the H_γ_ emission. The spatial evolution of luminescence behind the HCC is clearly observable for short HCC spanning 23 mm behind the nozzle electrode (water flow *Q* = 0.94 m^3^/h (15.7 L/min)). We observed a decaying luminescence tail spanning more than 5 cm behind the HCC collapse zone with clear asymmetry toward the bottom of the discharge tube. This asymmetry could be attributed to the discharge localization in the HCC due to locally asymmetric flow. The flow asymmetry originates from a slight off-axis alignment of the electrode in the Venturi nozzle. The most intense emission was observed in the active discharge region. However, the observed luminescence overlapped in this region with weak emission continuum (probably from the molecular hydrogen). The titanium emission lines resulting from the energetic processes at the electrode were also identified in the spectrum above the nozzle electrode. The radially integrated emission spectra resolved along the discharge axis revealed that (i) above the nozzle electrode region, the prevailing emission came from the discharge itself; (ii) in the cavitation region, the prevailing signal came from the luminescence and the ratio of luminescence intensity to the discharge emission intensity rose with the distance from the electrode. Only the luminescence emission was observed behind the cavitation collapse.

**Figure 2:**
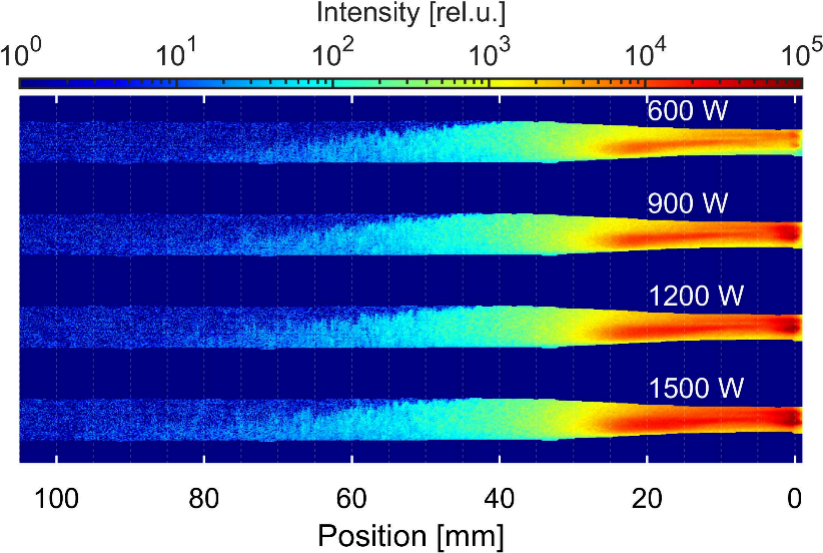
CaviPlasma discharge generated in alkaline Luminol/NaOH solution. The ICCD camera images for 420 nm wavelength, exposure time 33.3 ms. Labels represent HV input power. Flow rate was set to 0.94 m^3^/h; HCC length was 23 ± 4 mm. Flow direction from the right to the left.

To reveal the luminescence temporal dynamics and discuss the spatial distribution of the luminescence signal, temporally resolved imaging of the discharge-luminescence emission was performed using a fast-framing RGB digital camera at 1000 frames per second (exposure time 1 ms). The resulting sequence of frames is given in Figure 3. Extrapolating the available red, green, and blue channel sensitivity reported for the other Sony sensors equipped with a Bayer color filter^23–25^ we could assume the spectral representation of each color channel. The red channel (R) represents dominantly the H_α_ emission with a weak sensitivity shoulder also covering the luminescence. The intense narrow discharge channel could be identified on frame no. 7 with the maximum at the nozzle electrode (position 0 mm). Frame no. 8 represents the residual (luminescence) emission, and the exponential fit of emission decay reveals a rather fast emission decay of 0.5 ms.

**Figure 3:**
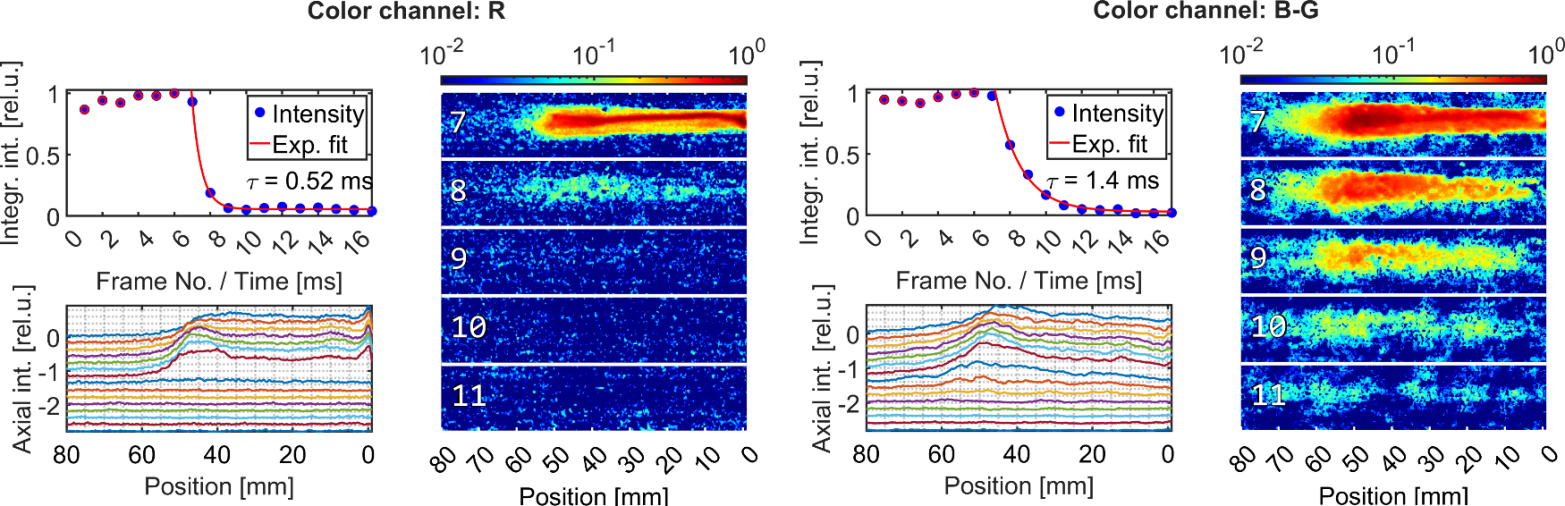
Fast-framing acquisition of discharge emission decay. The group of red color channel (R) represents dominantly the H_α_ emission, while the group of differential blue-green color channel (B-G) represents dominantly the luminescence emission. The top-left graphs represent the temporal evolution of the total emission intensity from 20 consecutive frames with 1 ms step and the exponential fit of emission decay after the active discharge phase. The corresponding relative intensity axial profiles for frame nos. 1–16 are given in bottom-left graphs; intensities from respective frames were intentionally shifted down in 0.1 units for better visibility, and the top profile represents frame no. 1 (time 1 ms). The right graphs represent 2D evolution of light emission for the selection of frame nos. 7 to 11 labeled in 1 ms steps. The input HV power prior to shutdown was 0.3 kW; the flow rate was 0.94 m^3^/h (15.7 L/min). The flow direction was from the right to the left. Frame exposure time: 1.0 ms.

The luminescence emission behavior could be followed using the difference of the blue and green channels (B-G) thanks to the estimated spectral sensitivities of these channels. The blue color channel dominantly represents the luminescence emission, but it is sensitive also to the H_β_ emission of comparable intensity. Fortunately, the reported green and blue channel sensitivities overlap near the H_β_ wavelength. Therefore, the difference of the blue and green image channels should represent similar spectral characteristics as the filtered ICCD images; compare frame no. 7 in Figure 3 to ICCD images in Figure 2. Moreover, as the luminescence decays at a much longer time scale, the intensity maps in frame no. 8 and subsequent frames should represent the sought discharge-free luminescence. The emission intensity evolution follows the exponential decay, and the fit of the integrated intensity gave us the estimation of effective luminescence lifetime to be 1.4 ms. This pseudo-first-order kinetics suggests the limited rate of postdischarge ROS–Luminol reactions. Thus, we could assume the luminescence emission intensity to be a marker of the ROS concentration profile in the active plasma zone.

The analysis of spatiotemporal distribution of luminescence decay is in agreement with the OH^•^ generation mechanisms in the literature.^11,14,26,27^ The electron-impact dissociation of water taking place in a water vapor-rich gaseous HCC environment leads to the formation of reactive species, dominantly the OH^•^ radicals.

For the luminescence mapping, these species must be transported to the liquid phase containing the Luminol chemical probe. This transport could happen either within the HCC, where the liquid in the form of mist could be present, or at the gas–liquid interface of the (nonlaminar) fast-flowing water stream surrounding the supercavitation cavity.

The spatiotemporal luminescence maps suggest that the OH^•^ transport to the surrounding water stream has to be considered to explain the observed behavior within the active plasma zone.

The maximum of luminescence at the HCC collapse region could be explained either as a result of a significant water stream velocity decrease at this region or as the collecting effect of OH-enriched mist from the supercavitation (discharge) region. The results in Odehnalova et al.^20^ suggest a sufficient OH^•^ lifetime for the latter mechanism. However, due to the fast-flowing water layer shielding effect, we do not have direct evidence for this mechanism.

However, the luminescence intensity maximum at the end of HCC could also be caused by the accumulation of Luminol in this highly stochastic region. In this region, the tempestuous phase transitions from the vapor to liquid occur, which could strongly enhance the ROS transport to the liquid phase and is therefore of great importance for the hydrodynamic cavitation plasma system performance.

A spatially resolved analysis must be performed to explore the luminescence dynamics further. Shirai et. al^9^ observed a less than 0.1 mm thick layer at the water surface from which the OH^•^-driven luminescence was observed. The length of the luminous tail behind the active discharge zone in CaviPlasma is, however, several tens of millimeters (see Figures 2 and 3). This suggests that the flow dynamics is involved in the observed long-range luminescence.

The spatial profiles of the luminescence decay were computed along seven adjacent axial streamlines in the discharge tube. The resulting profiles are given in Figure 4 for *Q* = 15.7 L/min (HCC length 23 mm) and applied power 1.2 kW. The curves in the figure represent decay profiles from seven 1.5 mm high horizontal strips (vertically integrated). The curves ordered from top to bottom represent the same order as in the maps in Figure 2. The intensity profiles (top-to-bottom) are vertically shifted by −2 orders for clarity.

**Figure 4:**
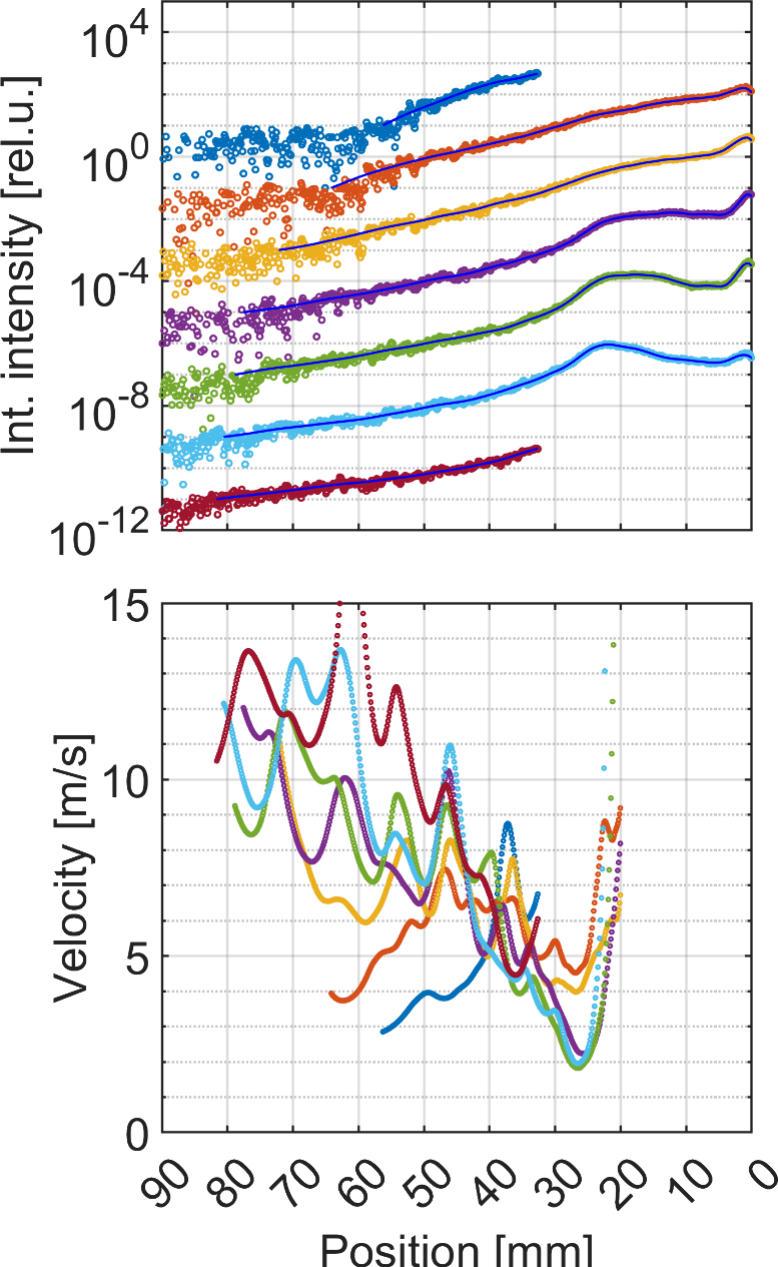
Top: Axial intensity profiles of discharge in Luminol/NaOH solution integrated across 7 vertical strips of 1.5 mm width (from top to the bottom of the discharge tube, the intensity profiles are vertically shifted by −2 orders for clarity). Bottom: The corresponding estimated stream velocity. Acquired using ICCD camera with optical interference filter at 420 nm, fwhm 10 nm. The HV power input was 1.2 kW at flow rate 0.94 m^3^/h (15.7 L/min). The flow direction was from the right to the left. Exposure time: 33.3 ms. The origin of the position axis represents the edge of the nozzle electrode.

Let us assume that the effective luminescence lifetime is constant in the flow behind the cavitation cloud collapse. Then we can correlate the spatial profile of the luminescence decay along the discharge tube axis to the local velocity of the liquid flow after the HCC collapse.

If the very simple model of radiative decay is assumed for the area behind the cavitation collapse and the transformation of the temporal variable to axial position using the local water stream velocity approximation with previously estimated effective luminescence lifetime (see Figure 3) is applied (eq 1), then the final formula could be derived by assigning local stream velocity (drift velocity) to the local luminescence intensity and its first spatial derivative at this point (eq 2).

1 $$-\frac{dI}{I}=\frac{dt}{\tau },,dt=\frac{dx}{v_{x}}$$

2 $$v_{x}=\frac{-I}{\tau \frac{dI}{dx}}$$

where *I* is the local luminescence intensity, τ is the effective luminescence lifetime, *x* is axial position, *v*_*x*_ is the velocity along *x* axis, and *t* is the time. This formula fitted onto the observed axial decay profile can be used to estimate the local flow velocity roughly. We performed this procedure on the luminescence data obtained using ICCD measurements. The following procedure was applied to calculate the first derivative of the noisy signal. The luminescence intensities were fitted using smoothing cubic splines with smoothing parameter *p* applied, obtaining input values of intensity *I* and its first spatial derivative to eq 2.

The result of this procedure for the seven integrated streamline intensities from the top to the bottom of the discharge tube is given in Figure 2. The streamlines have the thickness of 1.5 mm. The value (1 – *p*) = 1 × 10^–5^ was used for the fitting procedure representing a less-detailed, averaged perspective. The velocity model is valid only for the region after HCC collapse (position larger than 23 mm) as no additional source of luminescence is considered outside the active discharge zone.

The fully 2D resolved velocity profiles for input power of 1.2 kW and for 3 lengths of cavitation cloud corresponding to flow rates 15.7, 18.0, and 19.0 L/min are given in Figure 5. Before fitting with splines, the luminescence maps were vertically smoothed with the median filter of 0.5 mm width in order to decrease the noise in the data. To follow the subtle axial variations of luminescence, the finer spline smoothing parameter (1 – *p*) = 1 × 10^–6^ was used for 2D map computation. This approximation enabled the visualization of spatially localized inhomogeneities.

**Figure 5:**
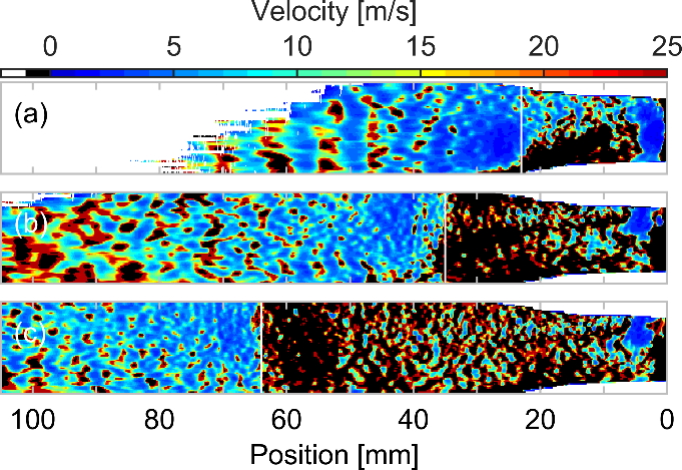
Reconstruction of stream velocity after HCC collapse based on the assumed luminescence decay rate constant τ = 1.4 ms. The comparison is given for 3 HCC lengths; see panels a–c. The applied power was kept at 1.2 kW at flow rates (a) 0.94 m^3^/h (15.7 L/min), (b) 1.08 m^3^/h (18.0 L/min), and (c) 1.14 m^3^/h (19.0 L/min). The white vertical lines show the end of HCC.

We can now compare the obtained 2D map of axial velocity estimated from the luminescence decay model (eq 2) with the flow dynamics estimations (see Figure 5). Although the flow downstream of the electrode is undoubtedly turbulent, which is valid also in the region behind HCC collapse, the plug flow model could be employed for comparison as a one-dimensional approximation of pipe flow. While it cannot capture the instantaneous velocity fluctuations inherent to turbulence, it can provide a reasonable approximation of the time-averaged turbulent velocity field, where the velocity profile tends to resemble a plug shape. Also one can expect the size of vortexes on the millimeter spatial scale. In this simplest solid-plug approximation the stream velocity is determined by the inner tube diameter and water flow rate. The plug flow velocity corresponds to 3–4 m/s for the flow rate in the range of 15–19 L/min and 10 mm inner diameter of tube. This is in a good agreement with the values obtained using the luminescence decay model (see Figure 5).

The resulting 2D velocity maps represent local drift velocity of the stream, and they give us insight into the stream dynamics. The decay model given by eq 1 is not valid in the active discharge zone. However, the model is sensitive to the places with positive luminescence spatial derivative, where the luminescence emission locally increases instead of anticipated decrease (decay), resulting in the negative values of calculated velocity. These places are represented as black spots in 2D maps given in Figure 5. However, this behavior enables us to distinguish between the active discharge zone and the decay zone behind the cavitation cloud, where the water stream surrounding the supercavitation collapses and has to slow down considerably.

The region of cavitation cloud collapse is also a region of high luminescence, signaling a high OH^•^ concentration in the liquid. The reason could be phase change from vapors in the cavitation cloud to liquid. The density rapidly increases around 4 orders of magnitude at the simultaneous pressure increase inside the collapsing cavities,^28^ resulting in the enhancement of OH^•^ transport into the liquid. Also the collecting effect of OH-enriched mist from the supercavitation (discharge) region could support the observed luminescence peak (compare Figures 3 and 4).

In the stream after the HCC collapse, the inhomogeneities occur on the 2D velocity maps in Figure 5. These inhomogeneities could represent the traces of undissolved gas bubbles remaining in the stream (see also the stream density simulation results in Cech et al.^12^). Moreover, the cavitation collapse represents a highly dynamic phase-transition phenomenon. Therefore, the shock waves could also be induced within the stream and possibly imprinted into the drift velocity field sensed using the chemical luminescence probe.

This study utilizes the Luminol chemiluminescence probe for the first time in the study of ROS in electric discharge generated in hydrodynamic cavitation. We can conclude that Luminol chemiluminescence reveals a significant concentration of the OH^•^ radical in the water surrounding the active discharge zone in HCC. The maximum of the luminescence signal was found in the collapsing end of the HCC. The further decay of the chemiluminescence behind the HCC end is caused by the transport of the excited Luminol probe by the water stream. A simple model based on the effective luminescence lifetime provides an estimation of the water drift velocity behind the HCC end.

## Experimental Section

The CaviPlasma unit adopted for the luminescence mapping experiment was described in detail in our previous diagnostics paper.^12^ Therefore, only a brief description will be given, and the setup differences from Cech et al.^12^ will be mentioned.

The hydraulic circuit consists of a water pump (CALPEDA MXHM 205/A, Calpeda S.p.A.; driven using a VYBO A550Plus Variable frequency driver; VYBO electric), a polypropylene reaction chamber (Venturi nozzle; 12 cm long, 12 mm outer diameter and 10 mm inner diameter, 7 mm constriction), and a 10 L reservoir connected using polyethylene 1 in. water pipes. No vacuum parts were adopted. The water flow rate was monitored by using a Keyence FD-H32 digital flow meter (Keyence International).

Electric discharge was ignited by using a custom-made high voltage (HV) power supply with an HV transformer. HV of 32 kHz frequency is imposed on a pair of electrodes positioned coaxially within the liquid stream. The nozzle electrode was made of titanium rod (4 mm in diameter, 5 mm incl. insulation) and positioned in the nozzle throat. The opposite ring electrode made of stainless steel tube (inner diameter of 10 mm) was placed 12 cm apart downstream of the reaction chamber after the HCC collapse region, so only the nozzle electrode was exposed to the gaseous environment and the discharge was generated against “liquid” electrode at the HCC collapse region; we labeled this regime as “unbridged” in Cech et al.^12^ The total average input power of the HV generator was measured using a Lumel ND10 watt-meter.

The optical emission of the system was probed spectrally using emission spectrometry (spectrometer Flame T, FLMT10459, OceanInsight) with quartz fiber and spatiotemporally using a digital camera (DSC-RX10 III, Sony Corporation) and intensified CCD camera (PI-MAX3, Princeton Instruments) equipped with a SIGMA 105/2.8 EX DG lens and optical interference filter at 420 nm (FBH420-10, Thorlabs).

The temperature of the treated liquid was measured using a Greisinger GTH 175 Pt1000 thermometer (GHM Group). The electrical conductivity of the treated liquid was followed by using the Mettler Toledo FP30 + LE703 probe.

For the luminescence mapping of ROS, the 5 L solution of 1 mM Luminol (98% purity, Acros Organics) and 0.1 M NaOH (Penta Chemicals) in deionized water was prepared for each experiment. The conductivity of the treated solution was 2.0 mS/cm. The solution temperature prior to treatment was 25 °C, and the temperature was 29 °C after the treatment. The pH of the solution was 10.5 with 5% estimated uncertainty.
